# Tailoring Type III Porous Ionic Liquids for Enhanced Liquid‐Liquid Two‐Phase Catalysis

**DOI:** 10.1002/advs.202401996

**Published:** 2024-03-14

**Authors:** Peiwen Wu, Bangzhu Wang, Linlin Chen, Jie Zhu, Ning Yang, Linhua Zhu, Chang Deng, Mingqing Hua, Wenshuai Zhu, Chunming Xu

**Affiliations:** ^1^ School of Chemistry and Chemical Engineering Jiangsu University Zhenjiang 212013 P. R. China; ^2^ College of Chemical Engineering and Environment State Key Laboratory of Heavy Oil Processing China University of Petroleum‐Beijing Beijing 102249 P. R. China; ^3^ School of Chemistry and Chemical Engineering Hainan Normal University Haikou 571158 P. R. China

**Keywords:** desulfurization, fuel oil, hydrophilicity, liquid‐liquid reaction, porous ionic liquids

## Abstract

Porous Ionic Liquids (PILs) have gained attention but facing challenges in catalysis, especially in liquid‐liquid two‐phase reactions due to limited catalytic sites and hydrophilicity control. This work engineered a Type III PILs (PILS‐M) using zeolitic imidazolate framework‐8 (ZIF‐8) confined phosphomolybdic acid (HPMo) as the microporous framework and N‐butyl pyridine bis(trifluoromethane sulfonyl) imide ionic liquid ([Bpy][NTf_2_]) as the solvent. The PILS‐M not only combines the advantages of traditional ionic liquids and microporous frameworks, including excellent extraction, high dispersion of catalytically active species, remarkable stability, *etc*., but also can make the inner surface of ZIF‐8 turned to be hydrophilic that favors the contact between aqueous hydrogen peroxide oxidant and catalytically active sites for the promotion of catalytic performance in reactive extractive desulfurization (REDS) processes of fuel oils. This study demonstrates Type III PILs' potential as catalysts for sustainable chemical processes, offering insights into versatile PILs applications in diverse fields.

## Graphical abstract

This work introduces innovative Type III Porous Ionic Liquids (PILS‐M), utilizing ZIF‐8 confined HPMo as the microframework and [Bpy][NTf_2_] as the steric solvent, to address catalytic challenges, offering a synergistic blend of traditional ionic liquids and microporous frameworks for enhanced catalytic performance in liquid‐liquid two‐phase reactions.

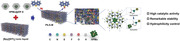

## 1 Introduction

Recently, a notable advancement has been achieved in porous liquids (PLs), which are liquids with permanent pores.^[^ ^1^ ^]^ The origin of PLs traces back to their inception by James et al. in 2007,^[^ ^2^ ^]^ and their experimental realization transpired concurrently by the same research group and Dai's group in 2015.^[^ ^3^ ^]^ Diverging from conventional porous solids, the distinctive essence of PLs manifests as an innovative subset of porous materials that perpetuate the enduring permanence of liquid porosity akin to their solid counterparts.^[^ ^4^ ^]^ Moreover, the intrinsic attributes of fluidic behavior, rapid heat, and mass transfer capabilities analogous to liquid dynamics, bestow upon PLs an expansive potential for multifaceted applications spanning diverse domains.^[^ ^5^ ^]^ Generally, PLs can be divided into four types,^[^ ^1^, ^6^ ^]^ and among the four distinct types of PLs, Type III PLs stand out as promising due to their straightforward synthesis strategy, customizable structures, and other advantageous features.^[^ ^5^, ^7^ ^]^

More recently, there has been a growing interest in porous ionic liquids (PILs), which have evolved from the foundational concept of PLs.^[^ ^1^, ^8^ ^]^ Type III PILs can be synthesized by simply dispersing porous frameworks, such as metal‐organic frameworks (MOFs),^[^ ^9^ ^]^ zeolite,^[^ ^7^, ^10^ ^]^ *etc*., into traditional ionic liquids (ILs), where the sizes of both cations and anions are larger than the pore sizes of the porous frameworks. Theoretically, Type III PILs can be designed by combining large‐sized ILs with small‐pore‐sized porous frameworks. Type III PILs combine the advantages of traditional ILs, such as fluidity and tunable structures, with those of porous solids, including porous structures and high specific surface areas.^[^ ^7^, ^11^ ^]^

However, it is worth noting that, to date, PILs, including Type III PILs, have primarily found utility in separation processes, such as gas capture and gas separation,^[^ ^1^, ^12^ ^]^ with only a limited number of studies concentrating on their potential applications in catalysis.^[^ ^7^, ^13^ ^]^ Expanding the applicability of PILs in catalysis holds substantial importance for broadening the utilization of PILs. Compared to conventional homogeneous catalysts, the pores within PILs notably enhance the dispersion of catalytically active sites. In contrast to traditional porous heterogeneous catalysts, pores in PILs maintain their openness, thereby enhancing mass transfer.^[^ ^13a^ ^]^ Furthermore, given the existence of multiple interfaces within PILs, it is envisaged that PILs possess the potential to rival homogeneous and heterogeneous catalysts. Although certain PILs have been employed in liquid‐gas two‐phase reactions,^[^ ^13^ ^]^ their utilization in liquid‐liquid reactions, particularly those involving both oil and water phases, such as reactive extractive desulfurization (REDS) of fuel oils with aqueous hydrogen peroxide (H_2_O_2_) as the oxidant,^[^ ^14^ ^]^ remains a challenge. This complexity arises from the inclusion of distinct liquid phases in such reactions, encompassing the oil phase, PILs phase, and H_2_O_2_ phase. The disparate hydrophilic properties require precise adjustments to the PILs to facilitate effective interaction between the oil phase and the H_2_O_2_ phase. Furthermore, it is important to note that many PILs lack sufficient catalytically active sites, which diminishes their catalytic activity in various reaction processes.

Further structural analysis of Type III PILs reveals a remarkable degree of adaptability within their porous framework structures, particularly in the context of MOFs‐based microporous frameworks. MOFs generally possess high specific surface areas, which can uniformly disperse catalytically active sites.^[^ ^15^ ^]^ Such MOFs supported catalysts not only can promote the catalytic performance but also reduce the consumption amount of catalytically active spices.^[^ ^16^ ^]^ However, the loss of catalytically active species during the catalytic process, particularly in “liquid‐liquid” two‐phase reactions, is unavoidable due to the weak interactions between the catalytically active species and MOF supports. Recent research endeavors have introduced an innovative “ship‐in‐bottle” strategy aimed at crafting MOFs‐based catalysts with enhanced stability.^[^ ^17^ ^]^ Through the utilization of this approach, catalytically active sites are typically confined within cages of MOFs.^[^ ^18^ ^]^ Inspired by these findings, if catalytically active species are confined within the microporous frameworks of Type III PILs, it offers several advantages. First, it prevents the loss of catalytically active species, ensuring their long‐term stability and recyclability. Additionally, the confinement of hydrophilic heteropolyacids can make the inner surface of MOFs turned to be hydrophilic, allowing H_2_O_2_ to enter the pores and react with the catalytically active sites during the liquid‐liquid reaction process.

As proof of such a concept, a Type III PILs (PILS‐M) was developed by incorporating zeolitic imidazolate framework‐8 (ZIF‐8) confined phosphomolybdic acid (HPMo) as the microporous framework (referred to as HPMo@ZIF‐8), and N‐butyl pyridine bis(trifluoromethane sulfonyl) imide ionic liquid ([Bpy][NTf_2_]) as the sterically hindered solvent. It was found that in the PILS‐M, the confining of HPMo in pores of ZIF‐8 favors the dispersion as well as the stability of HPMo, thereby promoting the REDS performance and preventing the loss of active sites. Additionally, the hydrophilic nature of HPMo enables the modulation of the inner surface hydrophilicity of ZIF‐8. Consequently, selective entry of the oxidizing agent H_2_O_2_ into the pores is facilitated. Such combined advantages induced remarkable desulfurization with sulfur removal of 100% and readily being recycled 8 times in REDS. This study not only provides an innovative strategy for creating stable PILs that exhibit excellent extraction and catalytic performance, but it also explores their application in the field of catalysis. These findings offer valuable insights into the potential utilization of PILs and broaden their scope for applications in various fields.

## 2 Results

### 2.1 Synthesis and Characterization of ZIF‐8 Confined HPMo

**Figure 1:**
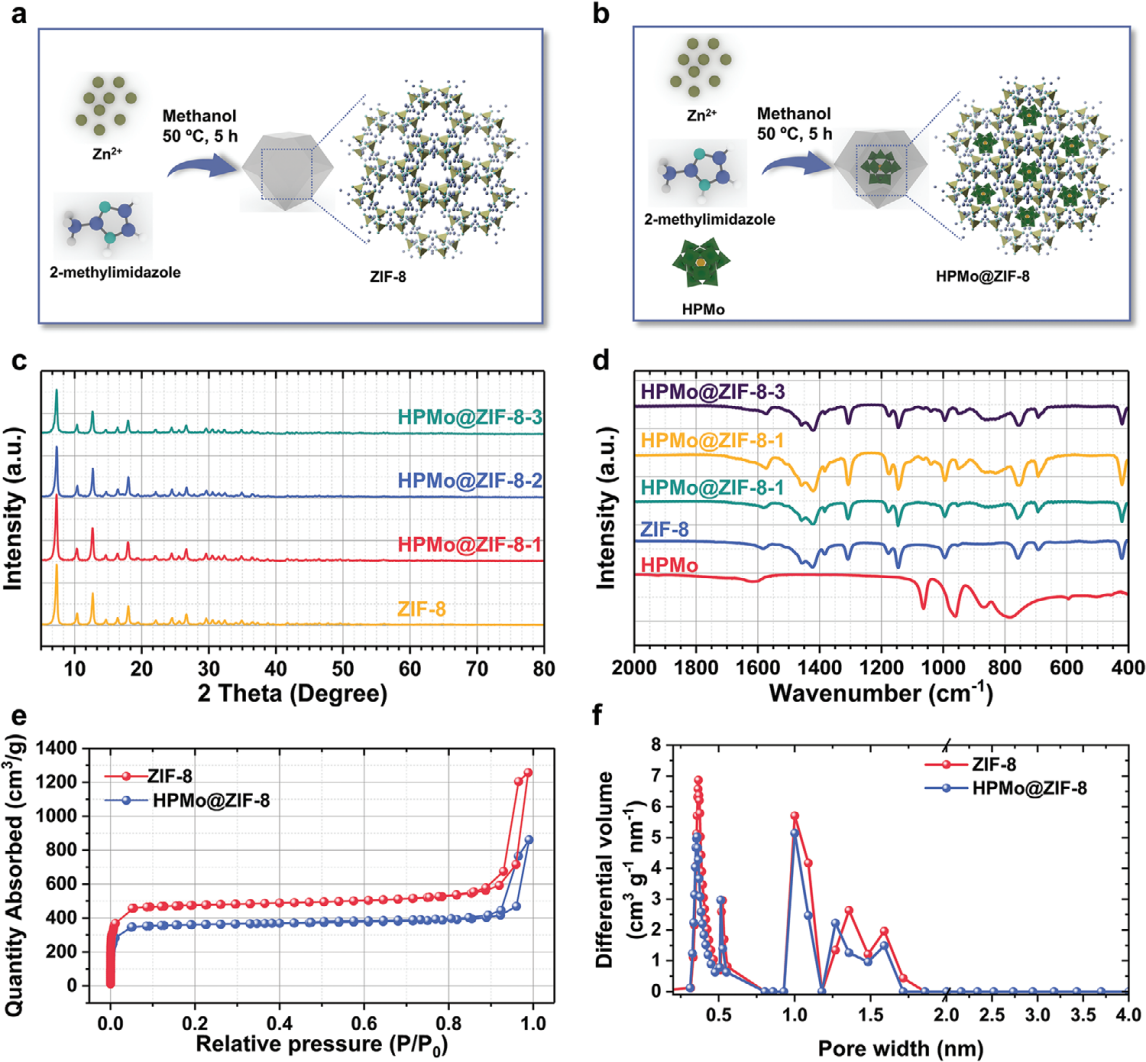
Synthesis and characterizations of HPMo@ZIF‐8 microporous frameworks. a) Schematic diagram for synthesis of ZIF‐8; b) schematic diagram for synthesis of HPMo@ZIF‐8 microporous frameworks; c) XRD patterns of ZIF‐8, and HPMo@ZIF‐8 microporous frameworks; d) FT‐IR spectra of HPMo, ZIF‐8, and HPMo@ZIF‐8 microporous frameworks; e) N_2_ adsorption‐desorption curves of ZIF‐8 and HPMo@ZIF‐8; f) Corresponding pore size distribution of ZIF‐8 and HPMo@ZIF‐8.

To construct Type III PILs, the pore size of the framework should be smaller than the cation and anion sizes of the IL, creating steric hindrance that restricts their entry. In this study, ZIF‐8 was chosen as the porous framework. To confine the catalytically active HPMo within ZIF‐8 (HPMo@ZIF‐8), a “ship‐in‐bottle” approach was employed to form the HPMo@ZIF‐8 framework. ZIF‐8 was synthesized by mixing zinc nitrate with 2‐methylimidazole in methanol, followed by magnetic stirring at 50 °C for 1 h (**Figure** **1**a). Meanwhile, for the synthesis of HPMo@ZIF‐8, all the steps were the same as those of ZIF‐8, except for the addition of varying amounts of HPMo in the methanol solution (Figure 1b). Samples were designated, based on the HPMo content, as HPMo@ZIF‐8‐1, HPMo@ZIF‐8‐2, and HPMo@ZIF‐8‐3. Unless otherwise specified, HPMo@ZIF‐8 represents the HPMo@ZIF‐8‐2 sample.

To verify the composition of the HPMo@ZIF‐8 microporous frameworks, X‐ray diffraction (XRD) patterns, Fourier Transform Infrared Spectroscopy (FT‐IR), and N_2_ adsorption‐desorption analysis were performed. It can be seen from Figure 1c that the diffraction peaks of the synthesized ZIF‐8 material closely match the reported diffraction pattern, and no impurities are detected.^[^ ^19^ ^]^ Furthermore, the XRD patterns of all HPMo@ZIF‐8‐x exhibit high similarities to that of ZIF‐8, suggesting that the confinement of HPMo does not significantly affect the framework structure of ZIF‐8. In addition, no distinct characteristic peaks for HPMo are observed, indicating the high dispersion of HPMo. This is primarily attributed to the relatively low crystallinity, low loading amount, and high dispersion of HPMo.

The structural characteristics of the prepared HPMo@ZIF‐8‐x microporous frameworks were investigated using FT‐IR spectra. As shown in Figure 1d, in the FT‐IR spectrum of HPMo, the characteristic peaks of the Keggin structure are evident. Meanwhile, In the FT‐IR spectrum of ZIF‐8, the absorption peak at 421 cm^−1^ and 990 cm^−1^ corresponds to the characteristic vibration of the Zn‐N bond and the characteristic vibration of the C‐N bond, respectively. With the confinement of HPMo, characteristic peaks for ZIF‐8 at 421 cm^−1^ and 990 cm^−1^ still can be detected, indicating the structural stability of ZIF‐8. Interestingly, the characteristic peaks corresponding to HPMo were found to be very weak and could only be detected in the HPMo@ZIF‐8‐2 and HPMo@ZIF‐8‐3, further confirming the high dispersion of HPMo.

The N_2_ adsorption‐desorption characterizations of ZIF‐8 and HPMo@ZIF‐8 at 77 K were carried out in Figure 1e,f. Both the prepared materials exhibit a rapid increase in N_2_ adsorption‐desorption curves at low relative pressures, followed by a stable region, which is a typical adsorption characteristic of microporous materials. Using the Barret‐Joyner‐Halenda (BJH) method, the specific surface area of ZIF‐8 was determined to be 1448 m^2^ g^−1^, while that of HPMo@ZIF‐8 was found to be 1100 m^2^ g^−1^. These results indicate the presence of abundant micropores within the porous frameworks. The decrease in specific surface area in HPMo@ZIF‐8 compared to ZIF‐8 provides further evidence of the confinement of HPMo within the pores of ZIF‐8. The pore size distribution analysis in Figure 1f, reveals the presence of numerous pores with sizes of 0.35 nm and 1.00 nm in ZIF‐8, which correspond to the cage sizes and cavity size of ZIF‐8, respectively. It is noteworthy that after confining HPMo, pores with pore sizes of ≈0.6 nm, 1.0 nm, 1.3 m, and 1.6 nm showed no obvious decrease. However, the 0.35 nm pores showed an obvious decrease, demonstrating that through the utilization of in‐situ assembly methods, HPMo molecules can be effectively confined within the cages ZIF‐8.

**Figure 2:**
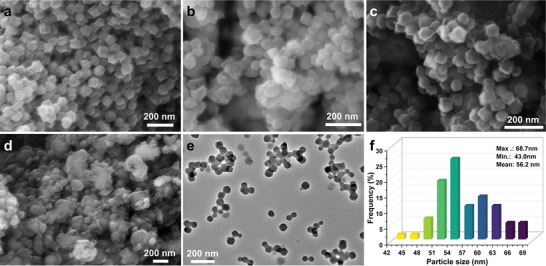
Morphology characterization of ZIF‐8 and HPMo@ZIF‐8 microporous frameworks. a) SEM image of ZIF‐8; b) SEM image of HPMo@ZIF‐8‐1; c) SEM image of HPMo@ZIF‐8‐2; d) SEM image of HPMo@ZIF‐8‐3; e) TEM image of HPMo@ZIF‐8; f) corresponding particle size distribution of HPMo@ZIF‐8 in Figure 2e.

Scanning electronic microscopy (SEM) characterization was performed to analyze the morphology and microstructure of HPMo@ZIF‐8 porous frameworks, and the results are displayed in **Figure** **2**. The results reveal that ZIF‐8 nanoparticles exhibit a hexagonal block‐like shape with a relatively low aggregation (Figure 2a). The grain size of ZIF‐8 is determined to be < 100 nm. Upon the introduction of HPMo, the morphology of HPMo@ZIF‐8‐1 and HPMo@ZIF‐8‐2 does not exhibit significant changes compared with that of ZIF‐8, but some degree of particle aggregation is observed (Figure 2b,c). As the HPMo loading increases, a serious particle aggregation is observed in the HPMo@ZIF‐8‐3, accompanied by the formation of cluster‐like structures (Figure 2d). This phenomenon can be attributed to the excessive addition of HPMo, which lowers the pH of the reaction solution, subsequently leading to the protonation of ligands and inhibiting the coordination of metal clusters. To gain further insights into the morphology of the HPMo@ZIF‐8, transmission electronic microscopy (TEM) characterization was conducted in Figure 2e. The TEM image reveals that the HPMo@ZIF‐8 porous framework particles exhibit a uniform distribution. Additionally, measurements of the nanoparticle size indicate an average size of 56.2 nm (Figure 2f). The small particle size of the porous framework is advantageous for the formation of a stable colloidal system.

### 2.2 Synthesis and Characterization of PILS‐M

**Figure 3:**
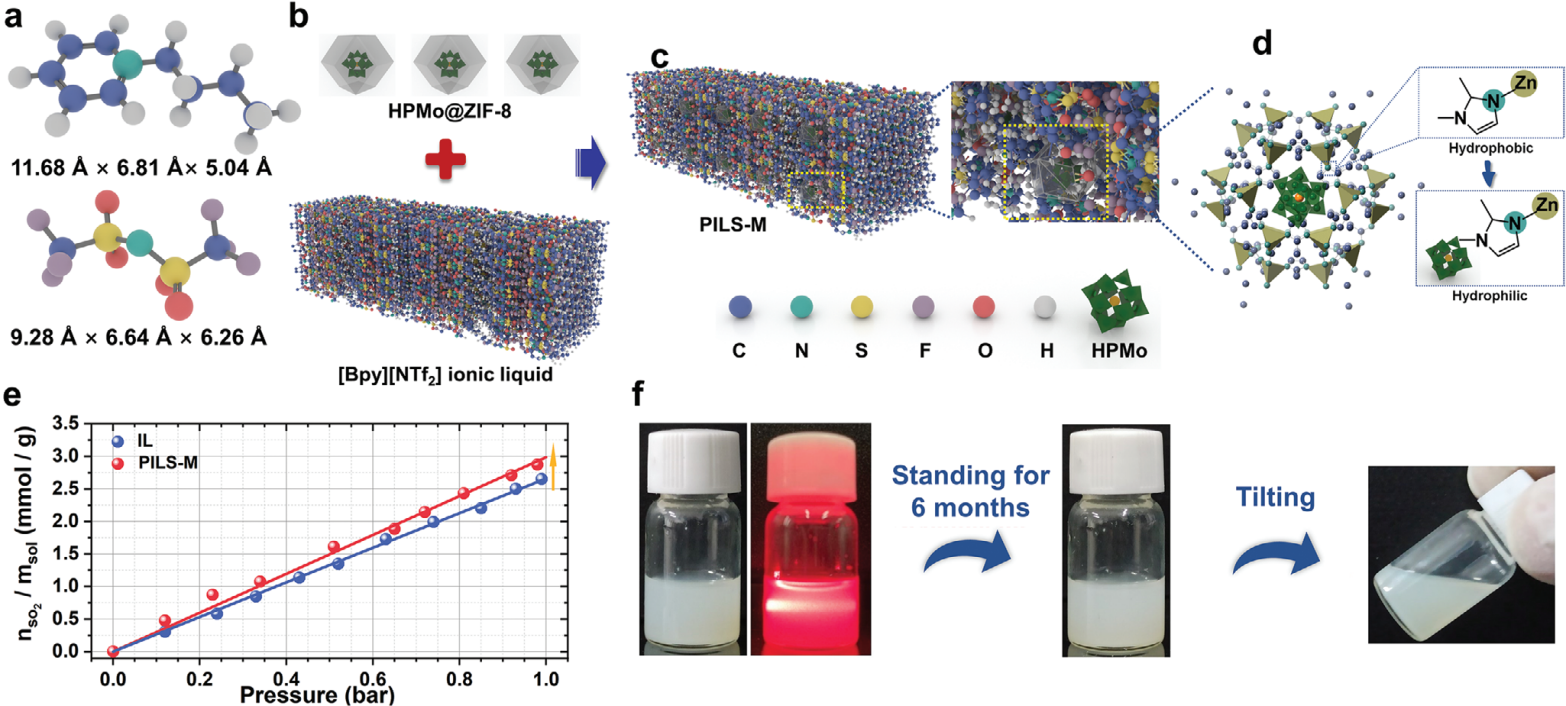
Synthesis and characterizations of PILS‐M. a) Calculated sizes of [Bpy]^+^ cation and [NTf_2_]^−^ anion; b) schematic diagram of HPMo@ZIF‐8 and [Bpy][NTf_2_]; c) schematic diagram of PILS‐M; d) schematic diagram of the confinement of HPMo by ZIF‐8, which tunes the hydrophilicity of inner surface of ZIF‐8; e) gas adsorption performance of IL and PILS‐M under different pressure with SO_2_ as the probing molecular; f) optical photographs of PILS‐M with Tyndall effect and optical photograph of PILS‐M standing for 6 months.

To engineer the Type III PILs, it was crucial to ensure that the pore size of the HPMo@ZIF‐8 porous framework is smaller than the molecular size of the [BPy][NTf_2_] organic guest. This size constraint allowed the cations and anions of the ILs to be blocked from entering the pores of the porous framework, preserving the original porous structure and resulting in PILs. The N_2_ adsorption‐desorption curve from the above analysis revealed that the nano‐cage size of HPMo@ZIF‐8 was measured to be 3.5 Å. Further computational analysis using B3LYP/6‐31g(d) level and Multiwfn software provided the molecular structure of the [Bpy][NTf_2_] in **Figure** **3**a. The analysis indicated that the size of the [Bpy]^+^ cation is 11.68 Å × 6.81 Å × 5.04 Å, and the size of the [NTf_2_]^−^ anion is 9.28 Å × 6.64 Å × 6.26 Å, showing that both the cation and anion are larger than the cage window size. As a result, the cations and anions of the [Bpy][NTf_2_] were blocked from entering the pores of the ZIF‐8. Therefore, the dispersion of HPMo@ZIF‐8 within [Bpy][NTf_2_] (Figure 3b) results in the formation of the PILs with permanent porous structures (Figure 3c). In addition, based on previous crystallographic data of [Bpy][NTf_2_],^[^ ^20^ ^]^ the size of [Bpy][NTf_2_] is found to be 11.19 Å × 12.45 Å × 12.46 Å, also smaller than both the cage size and cavity size of ZIF‐8, also indicating that the [Bpy][NTf_2_] cannot enter pores of ZIF‐8. Such a result also verifies the formation of PILs by dispersing ZIF‐8 in [Bpy][NTf_2_]. Thus, by using HPMo@ZIF‐8‐1, HPMo@ZIF‐8‐2, and HPMo@ZIF‐8‐3 as the microporous frameworks, three different PILs were obtained, being denoted as PILS‐M‐1, PILS‐M‐2, and PILS‐M‐3. The contents of HPMo in PILS‐M‐1, PILS‐M‐2, and PILS‐M‐3 are determined to be 1.24 wt.%, 2.48 wt.%, and 4.72 wt.%, respectively, by Inductively coupled plasma atomic emission spectroscopy (ICP‐AES). Unless specified, PILS‐M stands for the representee PILS‐M‐2. Due to the larger molecular size of HPMo compared to the pores of ZIF‐8, HPMo can be confined within ZIF‐8, preventing any loss. Additionally, the inner surface of ZIF‐8 is the coordination between Zn^2+^ and organic 2‐methylimidazole, exhibiting hydrophobicity (Figure 3d). During the REDS process with aqueous H_2_O_2_ as the oxidant, H_2_O_2_ cannot access the pores. However, with the confinement of HPMo, its strong hydrophilic nature induces a transformation in the inner surface of ZIF‐8, rendering it more hydrophilic (Figure 3d). Consequently, H_2_O_2_ gains access to the pores of ZIF‐8 and reacts with HPMo, generating active intermediates for desulfurization. This unique structure imparts the PILS‐M with both permanent pore structures and exceptional performance for REDS.

To confirm the formation of PILs, a series of characterizations were performed. The presence of the porous structure in the PILs contributes to increased spacing between molecules, leading to a more dispersed mass relative to the volume within the material. As a result, the densities of the PILs are generally lower compared to those of conventional ILs. Experimental density measurements using a density meter showed that the densities of the [Bpy][NTf_2_] and the PILS‐M are 1.449 and 1.426 g cm^−3^ at 25 °C, respectively. This significant difference in density further confirms the formation of PILs. In addition, the absorption and capture capacity of gas molecules in both the [Bpy][NTf_2_] and PILS‐M were investigated at different pressures using SO_2_ as a probing molecule. The experimental results, depicted in Figure 3e, demonstrate that PILS‐M exhibits a significantly higher absorption capacity compared to the pure [Bpy][NTf_2_]. This indicates that PILS‐M contains additional space for gas storage, confirming that the pores of the porous framework are not occupied by the IL.^[^ ^12b^ ^]^

Furthermore, molecular displacement experiments were conducted using small trichloromethane (CHCl_3_) molecules as guest species. The molecular dimensions of CHCl_3_ are comparatively smaller than the pore sizes of ZIF‐8, enabling facile ingress of CHCl_3_ molecules into the framework's pores. As shown in Figure S1 (Supporting Information), upon introducing CHCl_3_ into the PILS‐M followed by brief agitation, the emergence of tiny gas‐filled cavities can be observed within the structure. The initiation of this gas‐evolving phenomenon can be explained by molecular displacement mechanism rather than intrinsic alterations in the PILs, further confirming the presence of permanent micropores in the PILS‐M.

The dispersion stability of the PILs is a crucial performance factor. Aggregation of nanoparticles with the liquid matrix would lead to precipitation.^[^ ^21^ ^]^ To evaluate the dispersion stability, the precipitation method is employed, involving static settling to observe any sediment in the dispersion, indicating its stability. In Figure 3f, it is evident that the prepared PILS‐M sample is uniform, with no visible solid particles. The Tyndall effect can be observed when the sample is irradiated with a laser, confirming the formation of a stable colloidal system. This indicates that the HPMo@ZIF‐8 nanoparticles are highly dispersed in the [Bpy][NTf_2_]. Even after 6 months of standing at room temperature, the PILS‐M remains homogeneous with no precipitation, primarily attributed to the strong electrostatic interaction between the surface of HPMo@ZIF‐8 and the [Bpy][NTf_2_]. Additionally, the small particle size of HPMo@ZIF‐8, which is confirmed by SEM and TEM in Figure 2, contributes to the system's stability.^[^ ^22^ ^]^ In conclusion, the prepared PILS‐M demonstrates excellent dispersion stability.

**Figure 4:**
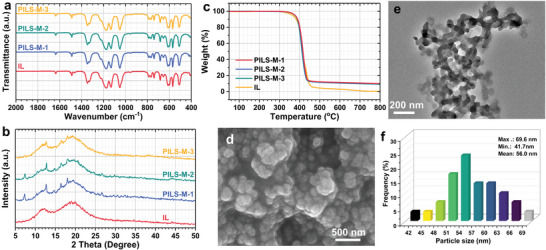
Characterizations of PILS‐M. a) FT‐IR spectra of IL, PILS‐M‐1, PILS‐M‐2, and PILS‐M‐3; b) XRD patterns of IL, PILS‐M‐1, PILS‐M‐2, and PILS‐M‐3; c) TGA curves of IL, PILS‐M‐1, PILS‐M‐2, and PILS‐M‐3; d) SEM image of PILS‐M‐2; e) TEM image of PILS‐M‐2; f) corresponding HPMo@ZIF‐8 particle size distribution of PILS‐M‐2 in Figure 4e.

Furthermore, additional characterizations were carried out in **Figure** **4**. The prepared PILs were subjected to FT‐IR in Figure 4a. In the FT‐IR spectrum of the pure [Bpy][NTf_2_], the characteristic peaks at 1172 cm^−1^ and 1050 cm^−1^ correspond to the S = O bond and S‐N‐S bond, respectively.^[^ ^23^ ^]^ Meanwhile, in the FT‐IR spectra of PILs, additional peaks are observed compared to the pure [Bpy][NTf_2_]. Specifically, peaks at 421 cm^−1^ and 990 cm^−1^ are observed, corresponding to the absorption peaks of the Zn‐N bond and C‐N bond, respectively, in ZIF‐8. Furthermore, the XRD patterns of [Bpy][NTf_2_], PILS‐M‐1, PILS‐M‐2, and PILS‐M‐3 are presented in Figure 4b. It is evident that the XRD pattern of the pure [Bpy][NTf_2_] exhibits two broad peaks. Notably, in the XRD patterns of PILS‐M‐1, PILS‐M‐2, and PILS‐M‐3, these characteristic peaks are still observable, and the characteristic peaks corresponding to HPMo@ZIF‐8‐x can also be identified. In addition, there is no discernible shift in the positions of all the characteristic peaks, suggesting that the structure of the porous solid framework is retained in the IL phase.

The thermal stability of the prepared PILs was assessed through thermogravimetric analysis (TGA) in Figure 4c. The TGA curve demonstrates that the PILs show minimal weight loss below 350 °C, indicating excellent thermal stability. SEM characterization was carried out to investigate the surface morphology of the synthesized PILs in Figure 4d. The image reveals the presence of dispersed solid particles with block‐like structures, indicating successful dispersion of the nano‐sized porous framework by [Bpy][NTf_2_]. Further characterization of the PILS‐M was performed using TEM in Figure 4e, revealing that the solid particles remain intact within the liquid medium. The average particle size of ≈56.0 nm (Figure 4f) shows no significant change compared with pristine HPMo@ZIF‐8 (Figure 2f), indicating high dispersion of the nano‐sized porous framework in the IL.

### 2.3 Catalytic Performance of PILS‐M

**Figure 5:**
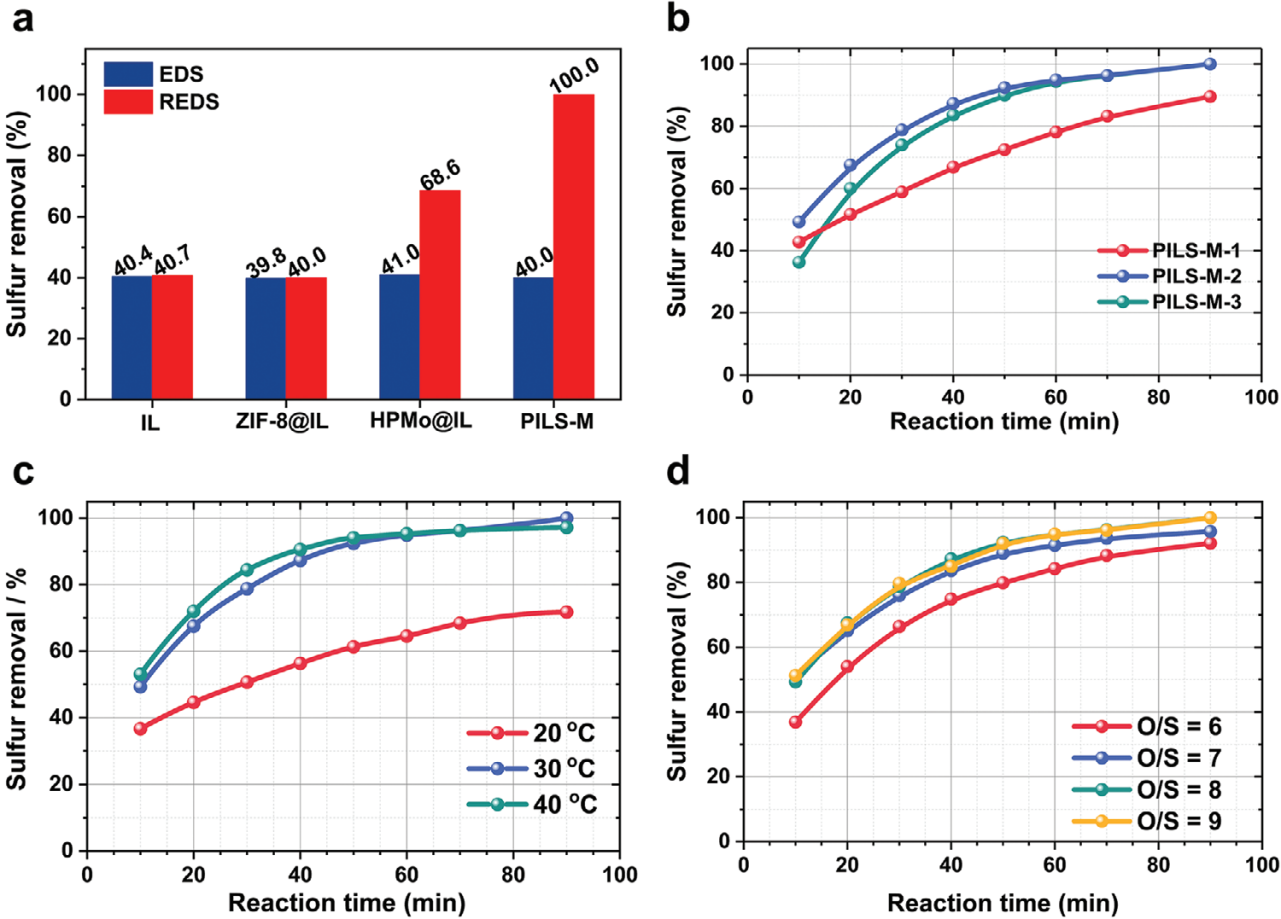
Desulfurization performances of different desulfurization systems. a) EDS and REDS performances of IL, ZIF‐8@IL, HPMo@IL, and PILS‐M; b) REDS activities of PILS‐M‐1, PILS‐M‐2, and PILS‐M‐3; c) the effect of reaction temperature on the REDS performance; d) the effect of oxidant amount on the REDS performance. In Figure 5a, ZIF‐8@IL stands for dispersing ZIF‐8 in [Bpy][NTf_2_], and HPMo@IL stands for dispersing HPMo directly in [Bpy][NTf_2_]. The contents of ZIF‐8 and HPMo in ZIF‐8@IL and HPMo@IL are the same as those in PILS‐M, respectively. The contents of [Bpy][NTf_2_] in ZIF‐8@IL and HPMo@IL are also the same as those in PILS‐M, respectively. The EDS conditions in Figure 5a: *V* (model oil) = 3 mL, *V* (IL or PILs) = 1 mL, *T* = 30 °C, *t* = 90 min; The REDS conditions in Figure 5a–d (expect changing the REDS parameter): *V* (model oil) = 3 mL, *V* (cat.) = 1 mL, *T* = 30 °C, *t* = 90 min, O/S = 8.

To emphasize the inherent advantages of PILs for REDS, we investigated the evaluative performance of various PILs and corresponding control samples in desulfurization systems using dibenzothiophene (DBT) as a model sulfur molecule in **Figure** **5**a. The analysis of the results reveals that the desulfurization efficiencies were ≈40% for various extractive desulfurization (EDS) systems. This outcome primarily arises from the effective extraction properties of [Bpy][NTf_2_] to DBT, indicating minimal influence exerted by solid inclusions on the EDS performance. Subsequently, H_2_O_2_ was employed as an oxidizing agent for REDS. Both [Bpy][NTf_2_] and [Bpy][NTf_2_]@ZIF‐8 do not exhibit significant improvements in desulfurization efficiency due to limited active sites. By employing HPMo as catalytically active sites within the IL phase (HPMo@IL), the desulfurization efficiency of the HPMo@IL was only 68.6%. This can be plausibly attributed to the aggregation of HPMo within the IL, thereby impeding effective dispersion. Notably, the complete removal of DBT was achieved by the PILS‐M. The exceptional extraction capacity of the IL results in a higher concentration of DBT within the PILS‐M phase. As discussed above, the pore size of HPMo@ZIF‐8 was measured to be ≈0.6 nm, which is smaller than the molecular size of DBT (≈0.8 nm) and larger than the molecular size of H_2_O_2_ (≈0.2 nm). In addition, the inner surface of HPMo@ZIF‐8 has been tuned to be hydrophilic. Thus, during the REDS process, the H_2_O_2_ can enter the pores of ZIF‐8 and is activated by highly dispersed HPMo to form activated intermediates for REDS.

The evaluation of REDS systems, being engineered with PILS‐M‐1, PILS‐M‐2, and PILS‐M‐3, which possess different loading amounts of HPMo, as shown in Figure 5b. It is worth noting that the desulfurization activity of PILS‐M‐1 is relatively lower, with a desulfurization rate of 89.5%. The decrease in REDES efficiency can be ascribed to the decreased amount of HPMo, which serves as an active component. After 90 min reaction, DBT is completely removed from model oils by both PILS‐M‐2 and PILS‐M‐3 as the reactive extractant. Nevertheless, upon detailed analysis, it becomes evident that during the first 60 min of REDS, PILS‐M‐2 exhibits higher efficiency when compared to PILS‐M‐3. The SEM images in Figure 2 reveal microporous frameworks with varying HPMo loadings, indicating that the porous framework of PILS‐M‐3 exhibits significant agglomeration. Excessive loading of HPMo results in pronounced aggregation within the porous framework, thereby hindering sufficient exposure of active sites. Based on these observations, this study chooses to proceed with further investigation using the PILS‐M‐2.

In practical industrial applications, the reaction temperature plays a crucial role as a pivotal parameter. The optimization of temperature for REDS can achieve a balance between energy consumption and desulfurization performance. Therefore, it is imperative to investigate the effects of reaction temperatures on the desulfurization system to determine the most favorable reaction temperature in Figure 5c. It can be seen from the result that the desulfurization efficiency is significantly low at a reaction temperature of 20 °C. This limitation stems from the inherent nature of the REDS mechanism, where the activation of H_2_O_2_ requires a specific amount of energy. In contrast, elevating the reaction temperature to 30 °C and 40 °C, significant improvements in desulfurization efficiency can be seen. It is noteworthy that the desulfurization efficiency decreased at 40 °C compared to that at 30 °C. This phenomenon can be attributed to the H_2_O_2_ self‐decomposition during the activation process of H_2_O_2_. Higher temperatures expedite the rate of self‐decomposition, resulting in reduced utilization efficiency of H_2_O_2_. To optimize desulfurization performance while conserving energy, a reaction temperature of 30 °C is considered the most optimized reaction temperature. Figure 5d investigates the effect of varying H_2_O_2_ dosages on sulfur removal. Notably, an incremental augmentation in the H_2_O_2_ amount leads to a progressive enhancement in REDS performance. The observed trend can be rationalized by the increased presence of activated species for sulfide oxidation, which is directly correlated to the higher H_2_O_2_ concentration. When the oxidant to sulfide molar ratio (O/S) raised from 6 to 8, there is an increase in REDS performance from 92.1% to 100%. It should be noted that further increasing the O/S only leads to marginal improvements in desulfurization efficiency. Therefore, an O/S of 8 is determined to be the optimized H_2_O_2_ dosage.

### 2.4 Confining Effect on Catalytic Performance

To emphasize the confining effect of HPMo by ZIF‐8, a control PILs sample was prepared using ZIF‐8 supported phosphotungstic acid (HPW/ZIF‐8) as the microporous framework and [Bpy][NTf_2_] as the solvent guests (denoted as PILS‐W). The N_2_ adsorption‐desorption curve and corresponding pore size distribution of the HPW/ZIF‐8 frameworks are presented in Figures S2 and S3 (Supporting Information). The results provide evidence for the presence of a relatively high specific surface area and a distinct microporous structure within HPW/ZIF‐8. However, it is pertinent to highlight that in contrast to the pore size distributions observed in Figure 1f for HPMo@ZIF‐8, a reduction in pores of varying pore sizes is discernible. The immobilization of HPW on the surface of ZIF‐8 results in the random blocking of ZIF‐8′s pores. Furthermore, Figure S4 (Supporting Information) presents the results of high‐angle annular dark‐field scanning transmission electron microscopy (HAADF‐STEM) images and corresponding elemental mappings for HPMo@ZIF‐8 and HPW/ZIF‐8. The HAADF‐STEM image in Figure S4a (Supporting Information) shows that HPMo@ZIF‐8 possesses a smooth surface without obvious particles. Elemental mapping in Figure S4b–d (Supporting Information) confirms the uniform distribution of HPMo within ZIF‐8, indicating that HPMo is confined within the cages of ZIF‐8.^[^ ^24^ ^]^ Conversely, the HAADF‐STEM image of HPW/ZIF‐8 (Figure S4e, Supporting Information) reveals numerous particles on the surface, suggesting that HPW is located outside ZIF‐8.^[^ ^24^ ^]^ Moreover, elemental scanning mapping in Figure S4f–h (Supporting Information) indicates an uneven dispersion of W.

The FT‐IR results OF PILS‐W depicted in Figure S5 (Supporting Information) provide further evidence for the simultaneous presence of HPW, ZIF‐8, and [Bpy][NTf_2_] within the PILS‐W. Additionally, the TGA analysis presented in Figure S6 (Supporting Information) demonstrates the exceptional thermal stability of PILS‐W, as evidenced by its negligible weight loss at temperatures below 350 °C. However, in the context of XRD characterization (Figure S7, Supporting Information), distinct from PILS‐M, a prominent presence of HPW peaks is evident in the XRD pattern of PILS‐W, indicating poor dispersion of HPW. Similarly, a gas adsorption experiment was conducted using SO_2_ as the probing molecule, with the outcome in Figure S8 (Supporting Information) revealing that PILS‐W exhibits an enhanced adsorption capacity compared to pure [Bpy][NTf_2_]. This result also confirms the porous structure of PILS‐W. Also, the optical photograph in Figure S9 (Supporting Information) shows that the PILS‐W also can remain stable after 6 months of standing at room temperature, verifying the formation of PILs.

Moreover, the PILS‐W was employed in the REDS of fuel oils, utilizing H_2_O_2_ as the oxidizing agent. Primarily, reaction parameters were optimized and are presented in Figures S10 and S11 (Supporting Information). These investigations reveal that the optimal reaction temperature is 60 °C, while the O/S was determined to be 6. Under these optimized conditions, PILS‐W demonstrates complete sulfur removal after 120 min reaction. Furthermore, comparative EDS and REDS performance analysis involving pure IL, ZIF‐8@IL, HPW@IL, as well as PILS‐W, was conducted in Figure S12 (Supporting Information). The results demonstrate the significantly superior desulfurization performance of PILS‐W, thereby emphasizing that the formation of PILS distinctly enhances the efficiency of REDS.

**Figure 6:**
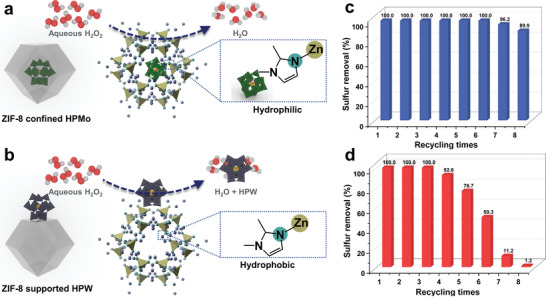
Structural schematic diagram of HPMo@ZIF‐8 and HPW/ZIF‐8 and the REDS stability of corresponding PILS‐M and PILS‐W. a) structural schematic diagram of HPMo@ZIF‐8; b) structural schematic diagram of HPW/ZIF‐8; c) REDS recycling performance of PILS‐M; d) REDS recycling performance of PILS‐W. The REDS conditions in Figure 6c,d: *V* (model oil) = 3 mL, *V* (IL or PILs) = 1 mL, *T* = 30 °C, *t* = 90 min, O/S = 8.

To understand the effect of confining effect of PILS‐M, both PILS‐M and PILS‐W were mixed with H_2_O, and then heated at 80 °C to evaporate additional H_2_O. Afterward, the PILS‐M and PILS‐W were characterized by FT‐IR in Figures S13 and S14 (Supporting Information). The discernible contrast of FT‐IR spectra between PILS‐M before and after the reaction reveals that, in comparison to the FT‐IR spectrum of PILS‐M, a novel peak at 3610 cm^−1^ corresponding to the ‐OH bond emerges in the FT‐IR spectrum of PILS‐M after mixing with H_2_O. However, for PILS‐W, no additional peak, especially the peak for ‐OH is detected. This result indicates that HPMo, a hydrophilic molecule, is confined within the internal pores of ZIF‐8 in PILS‐M, leading to modification of the inherent hydrophilicity of ZIF‐8′s inner surface. Consequently, this structure enables the penetration of the H_2_O_2_ oxidizing agent into the pores, thereby facilitating the reaction between HPMo and H_2_O_2_ to form active intermediates for REDS. Moreover, due to the larger molecular dimensions of HPMo compared to ZIF‐8′s pore size, effective retention of HPMo within the ZIF‐8 pores prevents undesired loss of catalytically active species (**Figure** **6a**). In contrast, PILS‐W exhibits a distinct behavior, where HPW is exclusively deposited on the surface of ZIF‐8. Although the surface loading of HPW in PILS‐W enhances HPW exposure, the solubility of HPW in water results in its dissolution in the aqueous H_2_O_2_ solution during the reaction. Consequently, loss of active components is inevitable in PILS‐W (Figure 6b).

Furthermore, the recycling performances of both PILS‐M and PILS‐W in the REDS system were assessed, as depicted in Figure 6c,d. The results distinctly illustrate the confinement effect of HPMo in PILS‐M preventing leaching during reaction. As a consequence, the desulfurization performance of PILS‐M remains to be above 90% even after the 8th cycle. Conversely, in the case of PILS‐W, the lack of robust active site retention results in a significant decline in desulfurization activity after the 4th cycle. This outcome further emphasizes that the confinement effect not only enhances the exposure of catalytically active centers but also contributes to stabilizing catalytically active sites, thereby enhancing the recycling performance.

## 3 Conclusion

In summary, we have developed Type III porous ionic liquids for liquid‐liquid heterogeneous catalysis by incorporating ZIF‐8 confined HPMo as the microporous framework, with [Bpy][NTf_2_] as the solvent matrix. The successful characterization of PILS‐M confirms the presence of the permanent porous structure. The confinement of HPMo within ZIF‐8 not only enhances the stability of catalytic active sites, preventing their loss but also regulates the hydrophilicity of ZIF‐8′s internal surface. This modulation facilitates the ingress of the H_2_O_2_ oxidant into the pores, enabling efficient contact and activation with the catalytic active sites. The application of PILS‐M for REDS of sulfur compounds from fuel oils demonstrated remarkable desulfurization efficiency, achieving 100% sulfur removal under optimized reaction conditions. Importantly, this strategy for designing Type III PILs proves to be universally applicable, as demonstrated through a series of comparative experiments. Overall, this study introduces an innovative design strategy for creating stable PILs with excellent extraction and catalytic performance, and it showcases their practical application in the field of fuel desulfurization. These findings offer valuable insights into the potential versatility of PILs and extend their applicability across various domains, promising advancements in sustainable and efficient chemical processes.

## 4 Experimental Section

### Materials

Zinc nitrate hexahydrate (Zn(NO_3_)_2_·6H_2_O, A.R.), methanol (A.R.), phosphomolybdic acid (denoted as HPMo, A.R.), phosphotungstic acid (denoted as HPW, A.R.), hydrogen peroxide (H_2_O_2_, 30 wt.%) were purchased from Shanghai Sinopharm Group Chemical Reagent Co., LTD. N‐butyl pyridine bis(trifluoromethane sulfonyl) imide ionic liquid ([Bpy][NTf_2_], 99%) was obtained from Lanzhou Aolike Chemical Co., LTD.. 2‐methylimidazole (A.R.), cetane (C_16_H_34_, A.R.), dodecane (C_12_H_26_, A.R.), tetradecane (C_14_H_30_, A.R.) were gained from Shanghai Aladdin Chemical Technology Co., LTD. Dibenzothiophene (DBT, 98%), 4‐methyldibenzothiophene (4‐MDBT, 97%), 4, 6‐dimethyldibenzothiophene (4,6‐DMDBT, 98%) were purchased from Merck Sigma‐Aldrich.

### Preparation of Porous Ionic Liquids

*Preparation of ZIF‐8 Confined HPMo Porous Frameworks*: ZIF‐8 confined HPMo porous frameworks, denoted as HPMo@ZIF‐8, were synthesized via the following procedure: Zn(NO_3_)_2_·6H_2_O (0.7380 g) and 2‐methylimidazole (1.6430 g) were individually weighed and dissolved in methanol (50 mL). The solutions were then mixed and stirred using a heating‐controlled magnetic stirrer at 50 °C for 1 h. During the stirring process, specified masses of HPMo (0.0125 g, 0.025 g, and 0.05 g) were introduced into the reaction mixture. After completion of the reaction, the mixture was naturally cooled to room temperature and subjected to centrifugation with a speed of 8000 rpm for 5 min to obtain the solid product, which was subsequently washed three times with fresh methanol (20 mL). Finally, the solid was dried in a hot air oven at 80 °C overnight. The resulting samples were labeled as HPMo@ZIF‐8‐x, where x represents the amount of phosphomolybdic acid added during the reaction (*x* = 1, 2, and 3 for HPMo amount of 0.0125 g, 0.025 g, and 0.05 g, respectively). Unless specified, HPMo@ZIF‐8 stands for the representative HPMo@ZIF‐8‐2 sample.

*Preparation of ZIF‐8*: ZIF‐8 was prepared using the same process as HPMo@ZIF‐8, with the exception that no HPMo was added during the synthesis process.

*Preparation of ZIF‐8 Supported HPW Porous Frameworks*: ZIF‐8 supported HPW porous framework, denoted as HPW/ZIF‐8, was synthesized by an impregnation method. Dispersing the synthesized ZIF‐8 nanoparticles (0.15 g) uniformly in methanol (50 mL) using ultrasonication. Next, add HPW (0.025 g) to the solution and stir it at room temperature for a duration of 2 h, with a stirring speed of 400 rpm. Following the stirring process, subject the mixture to centrifugation (8000 rpm for 5 min) to collect the solid product. Wash the solid with fresh methanol (20 mL) and subsequently dry it in a hot air oven at 80 °C overnight. The resulting solid material is designated as HPW/ZIF‐8.

*Preparation of Type III Porous Ionic Liquids (PILs) using ZIF‐8 Confined HPMo Porous Frameworks*: The preparation of the porous ionic liquid process involves adding the obtained HPMo@ZIF‐8‐x or HPW/ZIF‐8 porous framework (0.05 g) to [BPy][NTf_2_] (1 mL). The mixture is then subjected to alternate stirring and sonication at room temperature for 2 h to achieve a uniform solution. The resulting sample is a porous ionic liquid with a porous framework content of 3.33 wt.%. Depending on the specific porous framework used, it is designated as PILS‐M‐1, PILS‐M‐2, PILS‐M‐3, or PILS‐W. Unless specified otherwise, PILS‐M refers to the most representative PILS‐M‐2 sample.

*Preparation of Type III PILs using ZIF‐8 Supported HPW Porous Frameworks*: Preparation of Type III PILs using ZIF‐8 supported HPW porous frameworks a the same as the preparation procedure of PILS‐M, except using ZIF‐8 supported HPW as the porous frameworks and the sample was denoted as PILS‐W.

### Reactive Extraction Desulfurization (REDS) Process

*Preparation of Model Oil*: Various model oils were synthesized with an initial sulfur concentration of 200 ppm by dissolving sulfur compounds, specifically dibenzothiophene (DBT), 4‐methyl dibenzothiophene (4‐MDBT), and 4,6‐dimethyl dibenzothiophene (4,6‐DMDBT), in dodecane. Hexadecane was incorporated as an internal standard at a concentration of 4000 ppm.

*REDS Experimental Process*: A mixture consisting of PILs (1 mL) and model oil (3 mL) was introduced into a 40 mL two‐necked flask. The temperature of the system was regulated by a thermostatic water bath. A specific quantity of H_2_O_2_ was added, initiating the reaction under magnetic stirring at 800 rpm. At regular intervals, the oil phase (1 µL) was extracted and injected into a gas chromatograph to quantify the sulfur content. The desulfurization efficiency of the catalyst toward sulfur compounds in the model oil was determined using the following formula:

(1) $$\mathrm{Sulfur}\;\mathrm{removal}\;\left(\%\right)\;=\;\frac{initial\;\;sulfur\;\;concentration\;\;(ppm)\;-\;sulfur\;\;concentration\;\;after\;\;REDS\;\;(ppm)}{initial\;\;sulfur\;\;concentration\;\;(ppm)\;}\times \;100\%$$

*Extractive Desulfurization (EDS) Process*: In the EDS experiment, the procedure follows a similar approach to the previously mentioned REDS process, with the exception that using ionic liquid instead of PILs.

*Recycling of the PILs*: Following the completion of the desulfurization reaction, the upper layer of the model oil is carefully separated. The reaction flask is then placed inside a forced air‐drying oven set at 70 °C to eliminate any residual oil. Once dried, fresh model oil and H_2_O_2_ are introduced into the dried reaction vessel for subsequent cycling experiments. To enhance the catalyst's cycling performance, after each reaction, the catalyst phase is isolated and subjected to two extractions using tetrachloromethane. The catalyst phase is subsequently dried in a forced air‐drying oven. Once the drying process is completed, the catalyst phase is reintroduced into the reaction flask for the next cycle of experiments.

### Characterization

The X‐ray diffraction (XRD) patterns of all prepared samples are studied using the Bruker D8 X‐ray diffractometer from Germany. The surface morphology and microstructure of samples are observed using Japan Electron Co., Ltd.’s JSM‐7800F field emission scanning electron microscope (FESEM) and JEM‐2100(HR) transmission electron microscope (TEM) and high‐resolution TEM (HRTEM). Agilent Technologies 7890/5975C and 7890A gas chromatography‐mass spectrometry (GC‐MS) instruments are employed for the separation, qualitative, and quantitative analysis of sulfur concentrations in oils. The molecular structure and chemical bonds of samples are investigated by Fourier Transform Infrared Spectroscopy (FT‐IR) on a Thermo Electron's Nicolet Nexus 470 infrared spectrometer. The specific surface area and pore structure of samples are evaluated using Nitrogen adsorption‐desorption curves on a Micromeritics Instrument Corporation's ASAP 2460 surface area analyzer. The thermal properties and decomposition behavior of samples are studied by thermogravimetry (TG) using Germany's Netzsch Instrument Manufacturing Co., Ltd.’s Q200 TA differential scanning calorimeter and Netzsch STA 449C thermal analyzer. The density of samples is measured using Anton Paar GmbH's DMA 4500 M Anton Paar density meter from Austria.

## Conflict of Interest

The authors declare no conflict of interest.

## Supporting information

Supporting Information

## Data Availability

The data that support the findings of this study are available from the corresponding author upon reasonable request.
