# Supplementary material for: Tailoring Type III Porous Ionic Liquids for Enhanced Liquid‐Liquid Two‐Phase Catalysis
Source: Adv Sci (Weinh). 2024 Mar 14;11(18):2401996. doi: 10.1002/advs.202401996 (PMC11095146; doi:10.1002/advs.202401996)
Supplement: Supplementary file 1 — Supporting Information [file ADVS-11-2401996-s001.pdf]

ADVANCED SCIENCE
Open Access

Supporting Information

for *Adv. Sci.*, DOI 10.1002/advs.202401996

Tailoring Type III Porous Ionic Liquids for Enhanced Liquid-Liquid Two-Phase Catalysis

Peiwen Wu, Bangzhu Wang, Linlin Chen*, Jie Zhu, Ning Yang, Linhua Zhu, Chang Deng, Mingqing Hua, Wenshuai Zhu* and Chunming Xu*

Supporting information

Tailoring Type III Porous Ionic Liquids for Enhanced Liquid-Liquid Two-Phase Catalysis

Peiwen Wu, Bangzhu Wang, Linlin Chen,* Jie Zhu, Ning Yang, Linhua Zhu, Chang Deng, Mingqing Hua, Wenshuai Zhu, * Chunming Xu*

Assoc. Prof. Dr. Peiwen Wu, Mr. Bangzhu Wang, Dr. Linlin Chen, Mr. Jie Zhu, Mr. Ning Yang, MS Chang Deng, Assoc. Prof. Dr. Mingqing Hua, Prof. Dr. Wenshuai Zhu
School of Chemistry and Chemical Engineering, Jiangsu University, Zhenjiang, 212013, P.R. China
E-mail: wupeiwen@ujs.edu.cn; chenllujs@ujs.edu.cn; zhuws@cup.edu.cn

Assoc. Prof. Dr. Peiwen Wu, Prof. Dr. Wenshuai Zhu; Prof. Dr. Chunming Xu
College of Chemical Engineering and Environment, State Key Laboratory of Heavy Oil Processing, China University of Petroleum-Beijing, Beijing, 102249, P.R. China

Prof. Dr. Linhua Zhu
School of Chemistry and Chemical Engineering, Hainan Normal University, Haikou, 571158, P.R. China

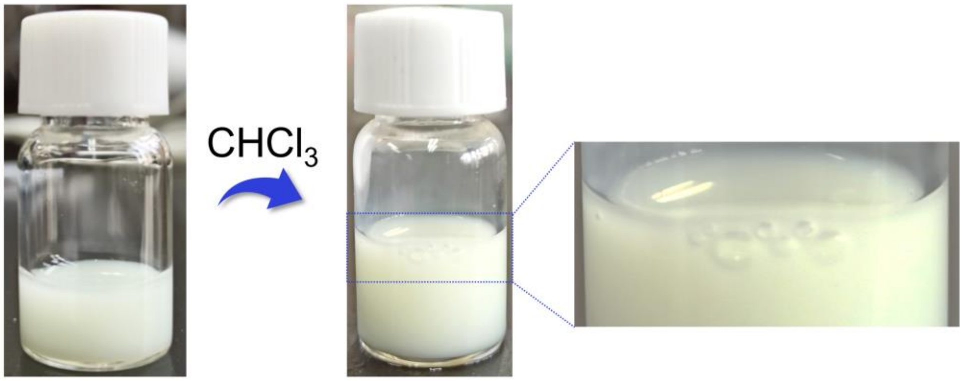

Figure S1. Optical photograph of the addition of a small guest molecule to PILS-M leads to the displacement of gas from the cavities and formation of bubbles.

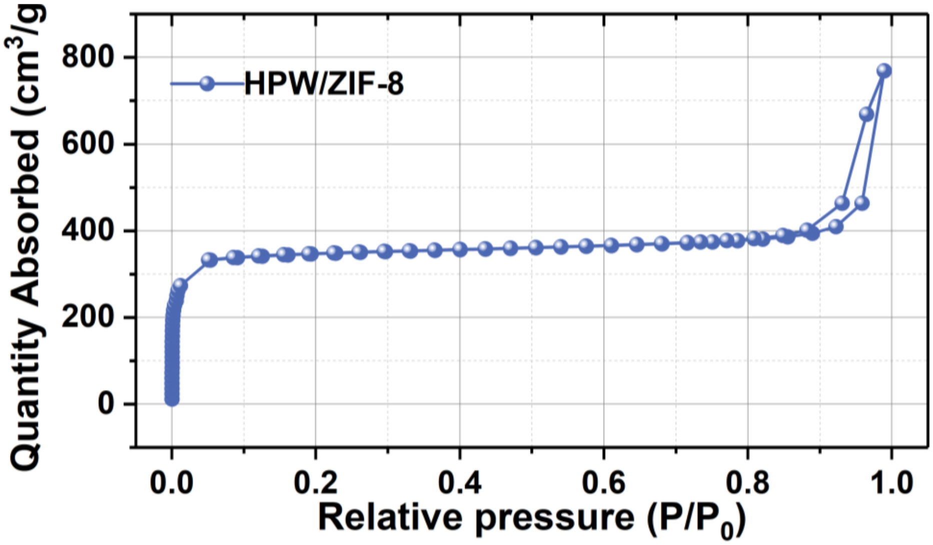

Figure S2. N_2 adsorption-desorption curve of HPW/ZIF-8.

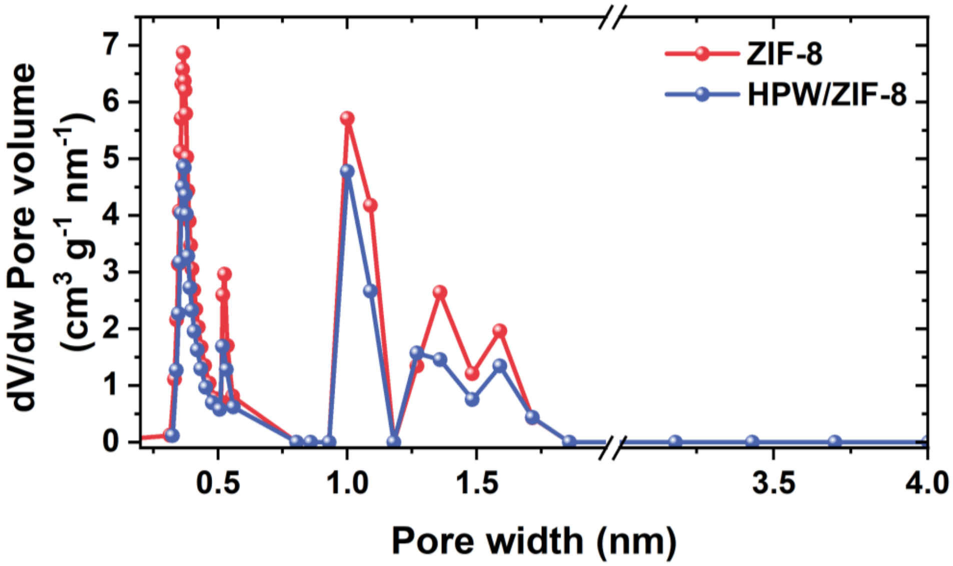

Figure S3. Corresponding pore size distribution of HPW/ZIF-8 and ZIF-8.

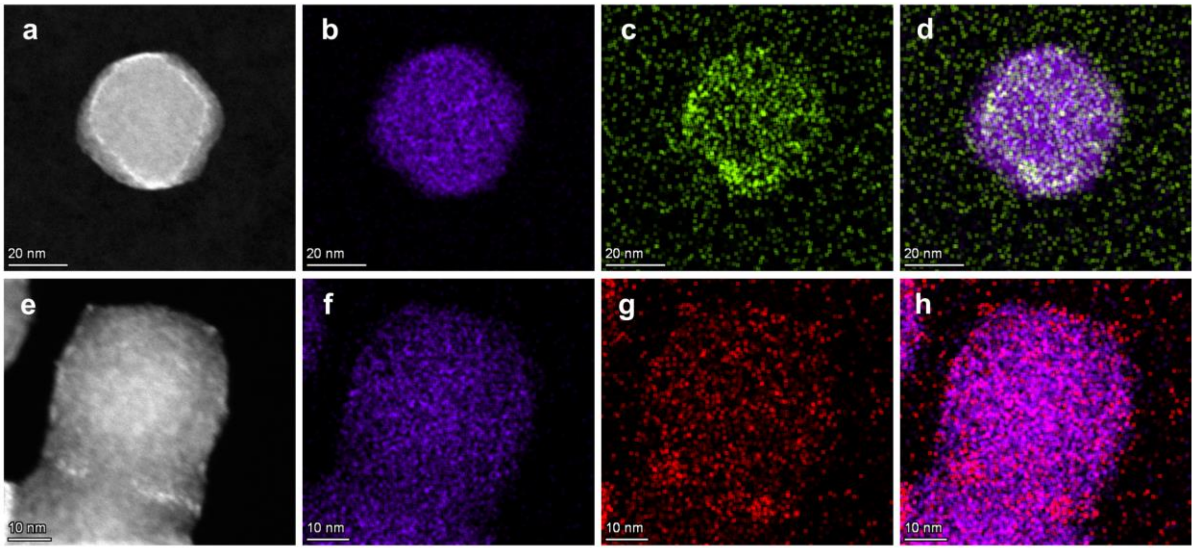

Figure S4. a) HAADF-STEM image of HPMo@ZIF-8, and b–d) the corresponding elemental mapping of Zn, Mo, and the overlapping elemental mapping of Zn, Mo. e) HAADF-STEM image of HPW/ZIF-8, and f–h) the corresponding elemental mapping of Zn, W, and the overlapping elemental mapping of Zn, W.

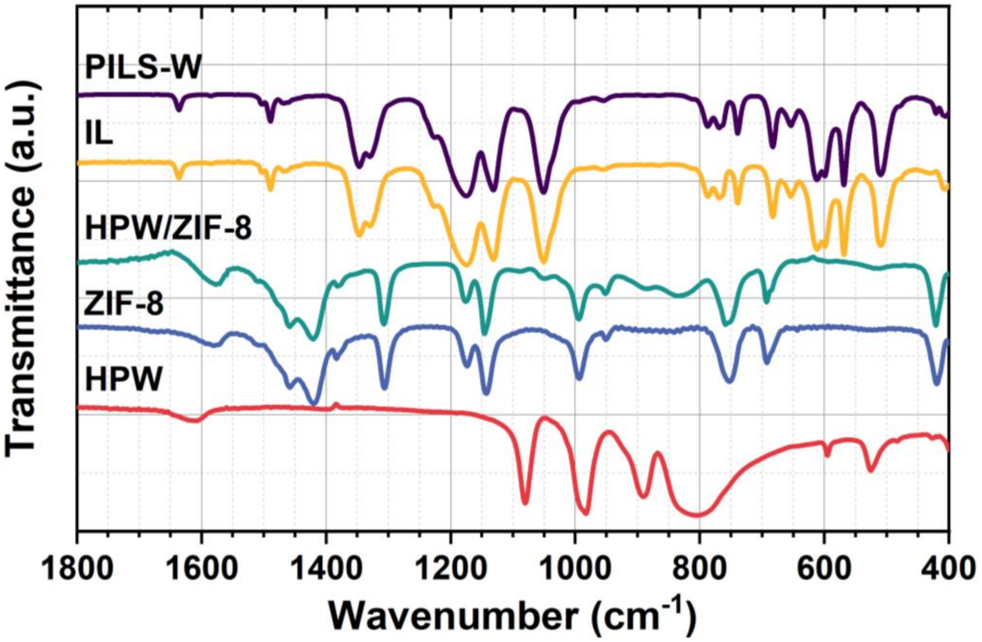

Figure S5. FT-IR spectra of HPW, ZIF-8, HPW/ZIF-8, IL, and PILS-W.

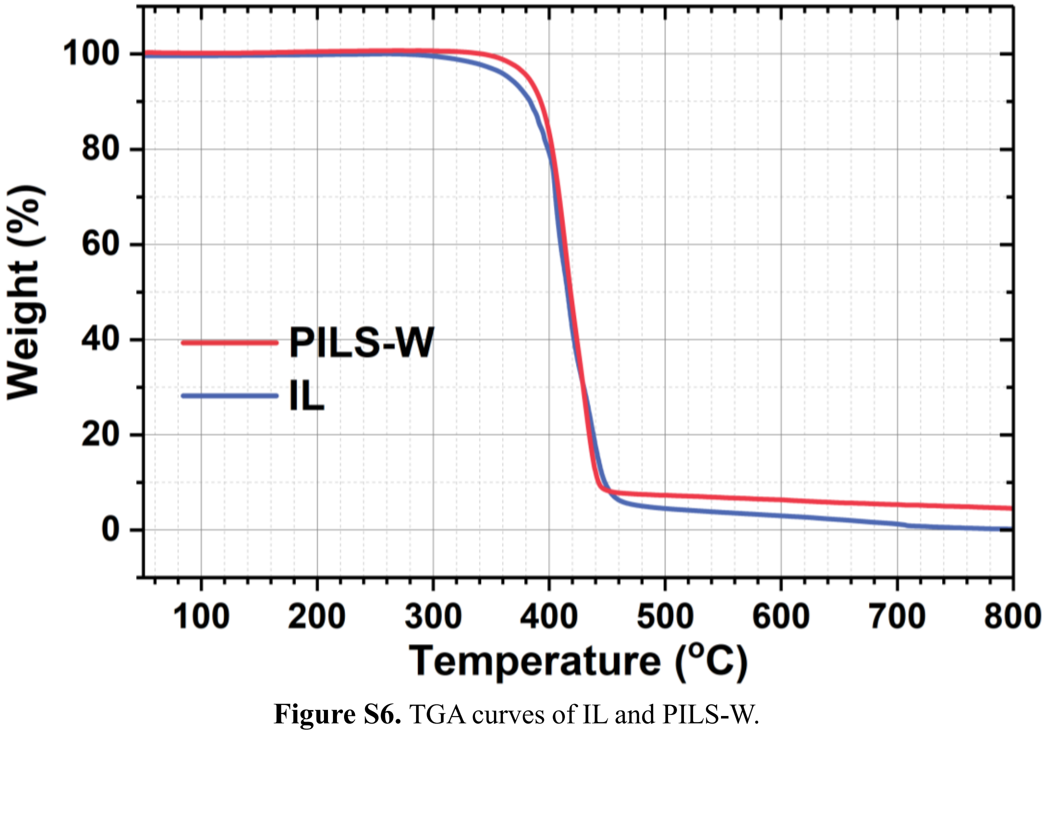

Figure S6. TGA curves of IL and PILS-W.

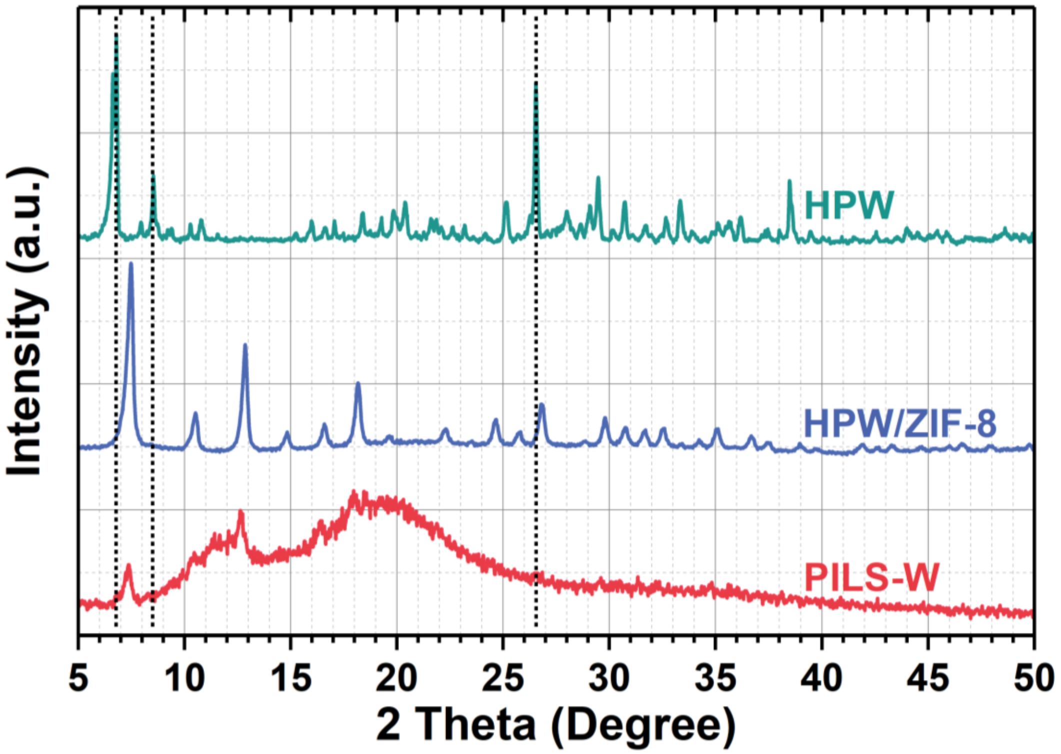

Figure S7. XRD patterns of HPW, HPW/ZIF-8, and PILS-W.

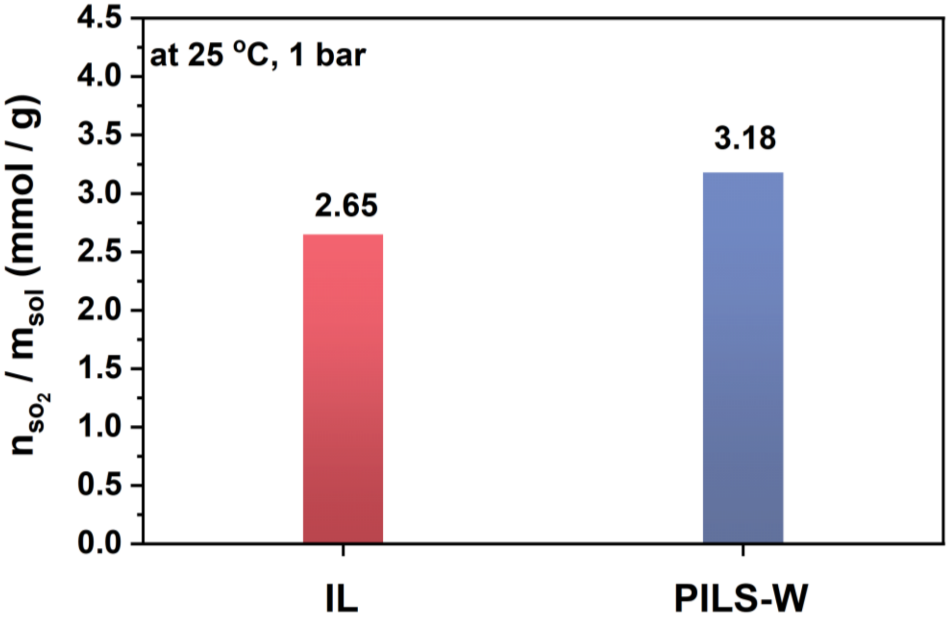

Figure S8. Gas adsorption performance of IL and PILS-W at 25 °C, 1 bar with SO_2 as the probing molecular.

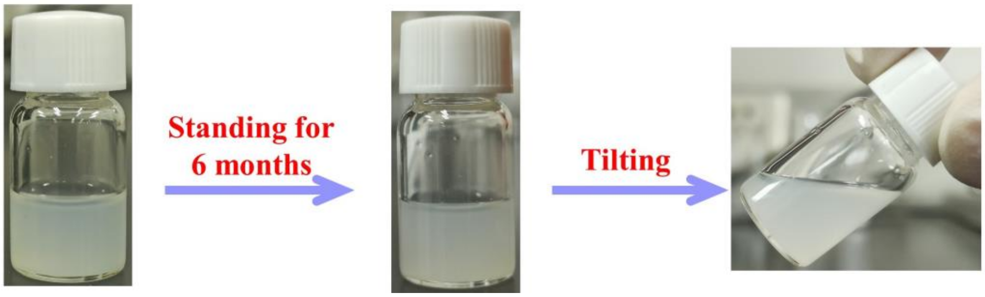

Figure S9. Optical photographs of PILS-W and optical photograph of PILS-M standing for 6 months.

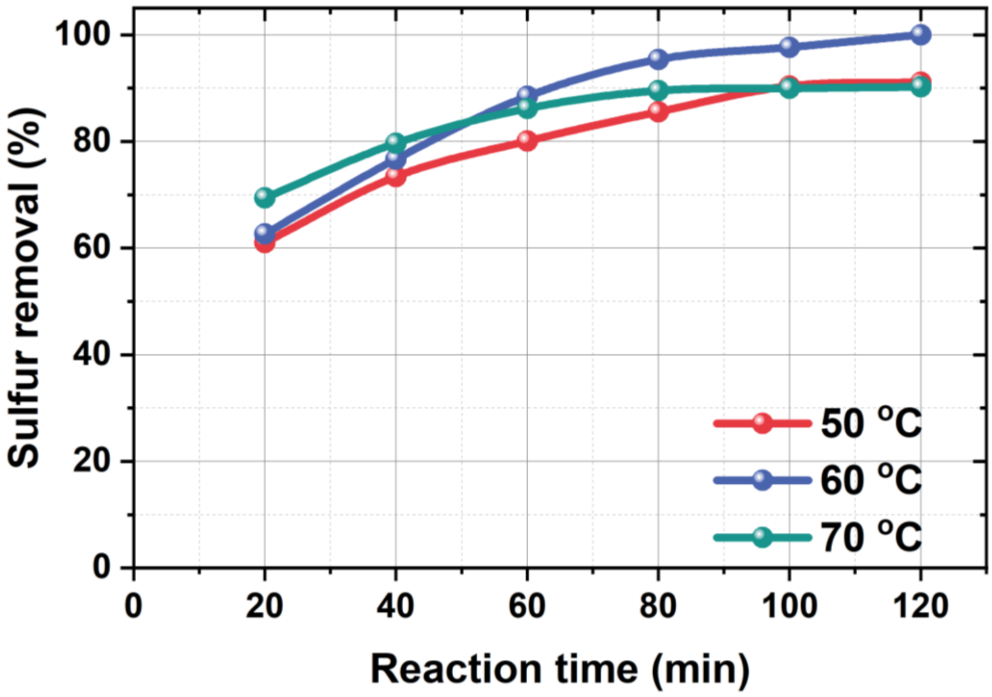

Figure S10. The effect of reaction temperature on the REDS performance.
The REDS conditions: V (model oil) = 3 mL, V (cat.) = 1 mL, O/S = 6.

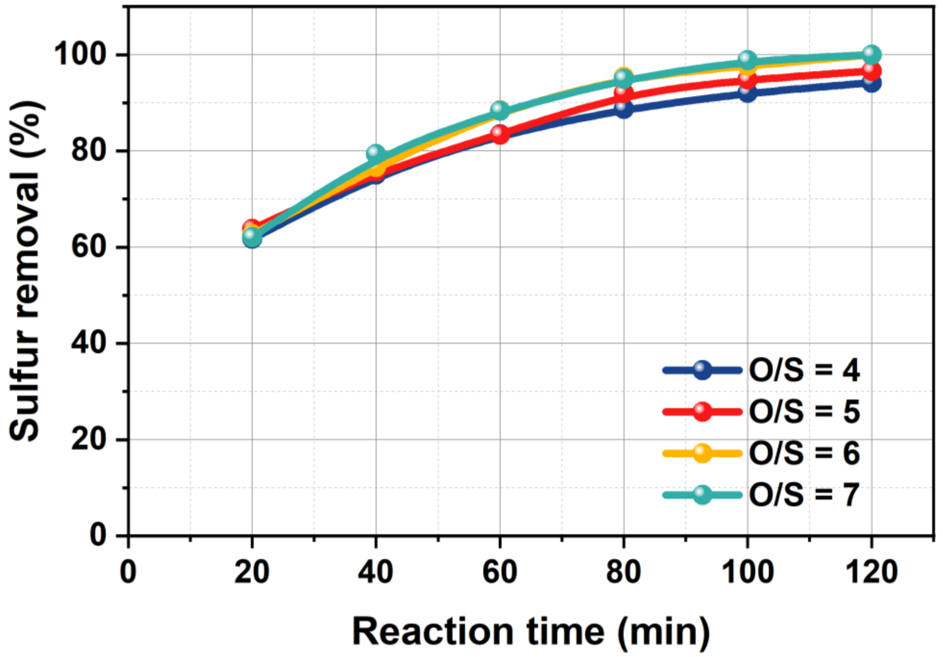

Figure S11. The effect of oxidant amount on the REDS performance.
The REDS conditions: V (model oil) = 3 mL, V (IL or PILs) = 1 mL, T = 60 °C.

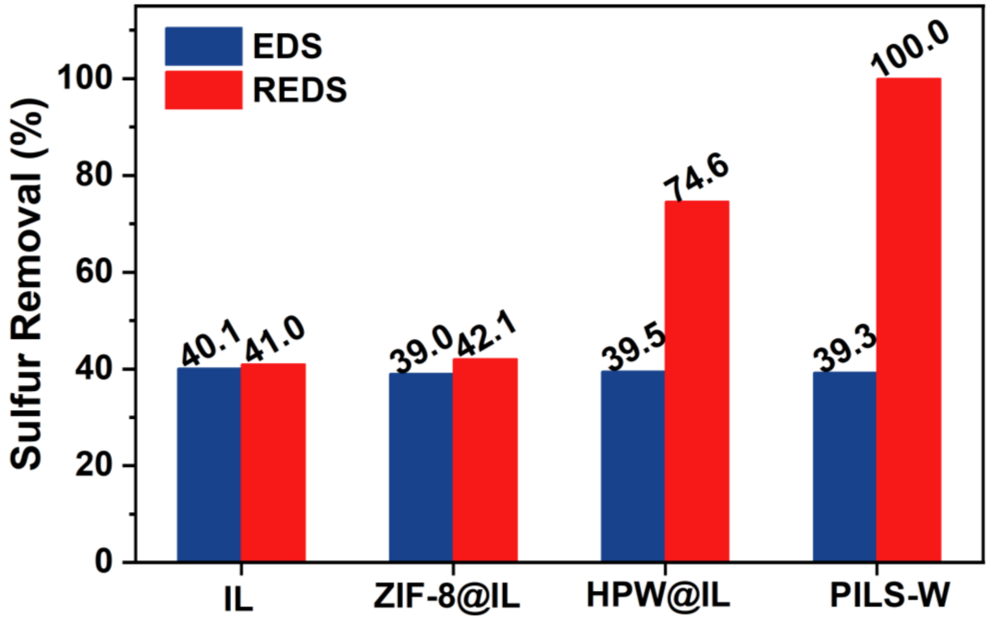

Figure S12 EDS and REDS performances of IL, ZIF-8@IL, HPMo@IL, and PILS-W**.**

In Figure S12, ZIF-8@IL stands for dispersing ZIF-8 in [Bpy][NTf$_2$], and HPW@IL stands for dispersing HPW directly in [Bpy][NTf$_2$]. The contents of ZIF-8 and HPW in ZIF-8@IL and HPW/IL are the same as those in PILS-W, respectively. The contents of [Bpy][NTf$_2$] in ZIF-8@IL and HPW@IL are also the same as those in PILS-W, respectively. The EDS conditions in Figure S12: V (model oil) = 3 mL, V (IL or PILs) = 1 mL, T = 60°C, t = 120 min; The REDS conditions in Figure S12: V (model oil) = 3 mL, V (cat.) = 1 mL, T = 60°C, t = 120 min, O/S = 6.

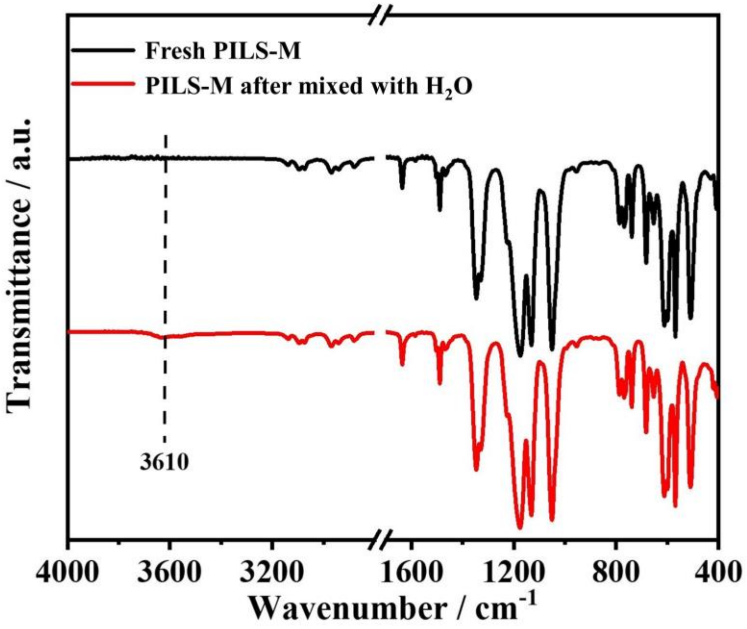

Figure S13 FT-IR spectra of PILS-M before and after being mixed with H_2O and sequent drying.

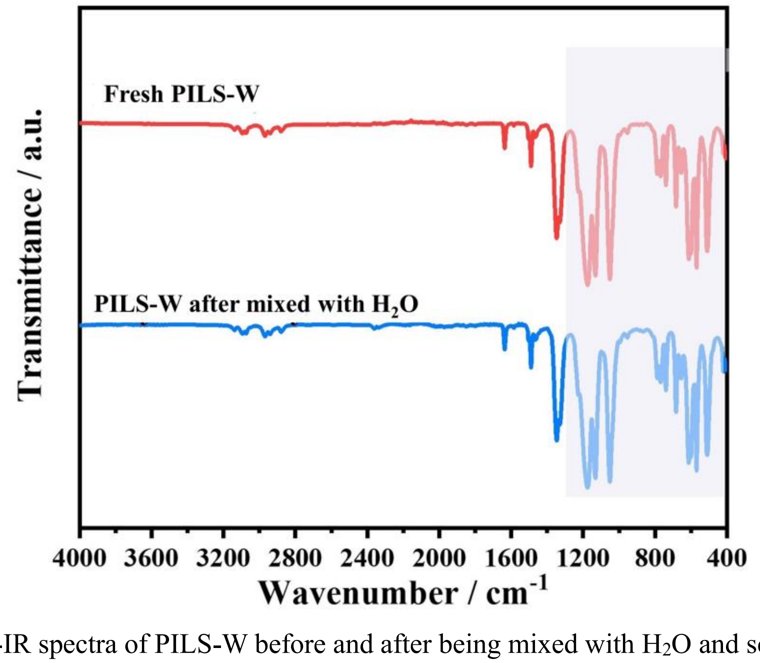

Figure S14 FT-IR spectra of PILS-W before and after being mixed with H_2O and sequent drying.
